# Absence of Rotation Perception during Warm Water Caloric Irrigation in Some Seniors with Postural Instability

**DOI:** 10.3389/fneur.2016.00004

**Published:** 2016-01-25

**Authors:** Elodie Chiarovano, Pierre-Paul Vidal, Christophe Magnani, Georges Lamas, Ian S. Curthoys, Catherine de Waele

**Affiliations:** ^1^CNRS UMR 8257, Cognition and Action Group, Centre Universitaire des Saints-Pères, Université Paris Descartes, Paris, France; ^2^ENT Department, Salpetriere Hospital, Paris, France; ^3^Vestibular Research Laboratory, School of Psychology, University of Sydney, Sydney, NSW, Australia

**Keywords:** semicircular canal, otolith, seniors, balance, video head impulse test, vertigo, vestibular neglect

## Abstract

Falls in seniors are a major public health problem. Falls lead to fear of falling, reduced mobility, and decreased quality of life. Vestibular dysfunction is one of the fall risk factors. The relationship between objective measures of vestibular responses and age has been studied. However, the effects of age on vestibular perception during caloric stimulation have not been studied. Twenty senior subjects were included in the study, and separated in two groups: 10 seniors reporting postural instability (PI) and exhibiting absence of vestibular perception when they tested with caloric stimulation and 10 sex- and age-matched seniors with no such problems (controls). We assessed vestibular perception on a binary rating scale during the warm irrigation of the caloric test. The function of the various vestibular receptors was assessed using video head impulse test (vHIT), caloric tests, and cervical and ocular vestibular-evoked myogenic potentials. The Equitest was used to evaluate balance. No horizontal canal dysfunction assessed using both caloric test and vHIT was detected in either group. No significant difference was detected between PI and control groups for the peak SPV of caloric-induced ocular nystagmus or for the HVOR gain. All the controls perceived rotation when the maximal SPV during warm irrigation was equal to or ≥15°/s. None of the subjects in the PI group perceived rotation even while the peak SPV exceeded 15°/s, providing objective evidence of normal peripheral horizontal canal function. All the PI group had abnormal Equitest results, particularly in the two last conditions. These investigations show for the first time that vestibular perception can be absent during a caloric test despite normal horizontal canal function. We call this as dissociation vestibular neglect. Patients with poor vestibular perception may not be aware of postural perturbations and so will not correct for them. Thus, falls in the elderly may result, among other factors, from a vestibular neglect due to an inappropriate central processing of normal vestibular peripheral inputs. That is, failure to perceive rotation during caloric testing when the SPV is >15°/s, should prompt the clinician to envisage preventive actions to avoid future falls such as rehabilitation.

## Introduction

Falls in seniors are a major public health problem. In studies of the risk of falls, seniors frequently report dizziness when walking; also standing upright in the dark becomes difficult with age (1). Maintaining balance for 30 s on a foam pad with eyes closed was impossible for 68% of healthy individuals over 70 years old (2). More than one in three people older than 65 years fall at least once a year (3). Falls cause primary injuries such as fracture or head injury. These lead to fear of falling, reduced mobility, and decreased quality of life in the long term (4, 5).

Falls have numerous causes, including deformations of skeletal geometry, peripheral, hind limb neuropathy, peripheral, foveal visual deficits, and vestibular deficits. Risk factors of falling are well described in the literature. The risk of falling increases linearly with the number of risk factors (3). Vestibular dysfunction is one of these risk factors (6, 7).

At the peripheral level, vestibular function changes with age. Horizontal canal function, as assessed by caloric stimulation (8) or video-HIT (9), does not appear to decline with age, but some reports of response to head impulses suggest that it does (10–12). In contrast, the use of ocular and cervical vestibular-evoked myogenic potentials (VEMPs) has provided evidence that otolith utricular and saccular functions are affected by age (13–16).

At the central level, findings concerning the changes to vestibular perception with age are consistent: there is no effect of age on self-motion perception (17, 18), but there is an increase in the variability of the perception threshold (19). In that context, it is also interesting that there is a very large literature on canal-otolith interaction – demonstrating that modulating otolithic input modifies canal-induced nystagmus and also canal-induced subjective sensations (20, 21). The neural basis for that interaction is also well established – convergence of otolith neurons onto second order canal neurons (22, 23). Finally, age seems to decrease the activation of the cortical area activated by caloric stimulations such as the ipsilateral parieto-insular vestibular cortex (PIVC) (24, 25).

The relationship between objective measures of vestibular responses and age has been studied. However, to our knowledge, the effects of age on vestibular perception during caloric stimulation and on the relation between the absence of vestibular perception and falls in senior subjects have not been studied. We therefore studied these issues in two groups of senior subjects: one reporting unstable feelings and exhibiting absence of vestibular perception when they tested with caloric stimulation and the other (age-matched) group with no such problems. Patients with poor vestibular perception may not be aware of postural perturbations, and so will not correct for them; such individuals may be more likely than their age-matched peers to fall. Thus, falls in the elderly may result from a vestibular neglect due to an inappropriate central processing of normal vestibular peripheral inputs.

We assessed vestibular perception on a simple binary rating scale during the caloric test and more particularly during warm irrigation. The function of the various vestibular receptors was assessed using vHIT, caloric tests, and cervical and ocular VEMPs (26). The Equitest was used to evaluate balance.

## Materials and Methods

Twenty subjects were included in the study: ten patients and ten controls:

Ten senior patients (six females, four males; mean age 77 ± 8 years; min–max: 66–85) were selected using two criteria:

- First, they complained of postural instability (PI): these patients reported feeling unstable as if they had drunk too much but without having consumed alcohol. They had to walk close to a wall if they wanted to walk in a straight line and reported feeling as if they were on a rocking boat.
- Second, these patients with PI complaints also displayed an absence of rotatory perception during warm caloric nystagmus. As it turned out, they had objectively measured PI greater than age-matched controls.

Ten age- and sex-matched seniors (mean age of 74 ± 6 years; min–max: 67–85) were also investigated. They did not complain of PI (controls).

The inclusion criterion for both groups was that the peak of the slow-phase eye velocity of their caloric nystagmus during warm irrigation should exceed 15°/s, providing objective evidence of normal peripheral horizontal canal function. Subjects were not included if they experienced vertigo or if they had a chronic inner ear disease (such as Meniere’s disease, positional vertigo, or vestibular neuritis), neurological problem, or abnormal MRI. All the patients were informed about the different vestibular and balance tests and gave written informed consent. The clinical Research Ethics Committee approved this work, registered at ANSM (ID RCB 2014-A00222-45).

### Dizziness Handicap Inventory

The Dizziness Handicap Inventory (DHI) questionnaire developed by Jacobson and Newman (27) reports activity limitation and restriction resulting from dizziness and unsteadiness. All subjects completed the DHI.

### Caloric Test

Caloric tests were performed using open-loop sequential bithermal external auditory conduct irrigations with water at 30 and 44°C and using video-nystagmography (Synapsis, France). The peak velocity of the slow phase of the induced-ocular nystagmus (peak SPV) was recorded for each warm and cold stimulation (30 s of irrigation) and for each ear. Percent canal paresis (CP) was calculated using Jongkees’ formula (28): CP = 100 × [(LW + LC) − (RW + RC)]/LW + LC + RW + RC, where LW, LC, RW, and RC are maximum velocity of the induced ocular nystagmus obtained on the left (L) and right (R) sides, with warm (W) and cold (C) water. A CP value above 25% was considered to indicate an abnormal response.

**Figure 1:**
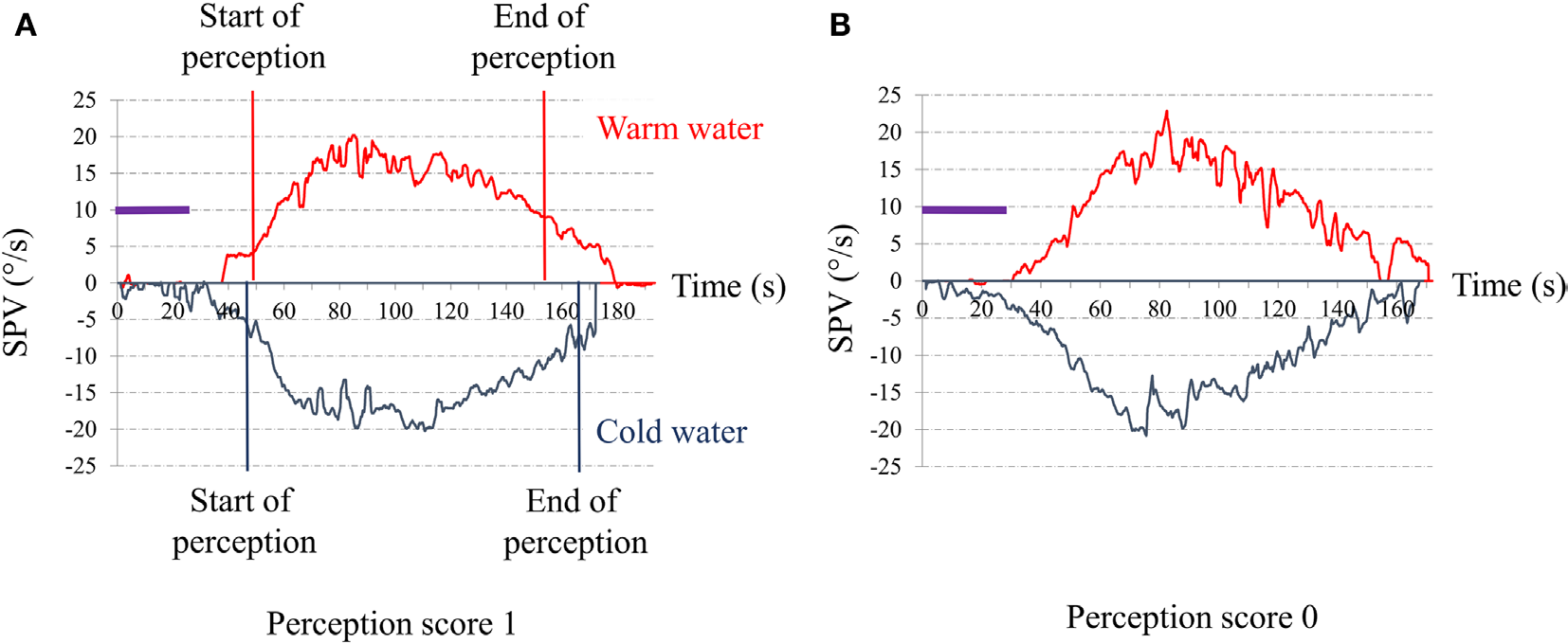
**Time series of the slow-phase velocity (SPV) of caloric nystagmus to warm and cold external canal ear irrigation**. Abscissa: time in seconds from the beginning of irrigation; Ordinates: SPV of the induced ocular nystagmus in degree per second; purple bar: duration of the ear irrigation (30 s). **(A)** Typical response of a control senior. The red vertical lines indicate the start and the end of the perception of rotation for warm irrigation. The blue vertical lines indicate the start and the end of the perception of rotation for warm irrigation. The perception appeared when the SPV reached a value for SPV close to 5°/s (start) and disappears when SPV decline to or below 10°/s (end). **(B)** Typical response of one of our senior complaining for postural instability. Note that despite the high SPV (exceeding 15°/s) to both warm and cold irrigations, the patient did not report any rotation perception.

For vestibular perception, we asked the subject to report feelings of rotation and/or dizziness during the post-warm-irrigation period while the induced caloric nystagmus was present. Vestibular perception was scored 1 if there was perception of body rotation whose direction (to the right or the left) could be clearly given by the patient (Figure 1A) and 0 if there was no perception of body rotation (Figure 1B).

We assessed perception of rotation to warm irrigation only because, in our patients, warm irrigation induced more vigorous ocular nystagmus than cold irrigation. Also, the peak SPV needed to be ≥15°/s, the value which induced a perception of rotatory vertigo in 100% of the controls. Individuals with a poor ocular response (SPV <15°/s) to warm caloric stimulations were excluded from the study.

### Video Head Impulse Test

Horizontal video-HIT (OtosuiteV^®^, GN Otometrics, Denmark) was used to test horizontal semicircular canal function (29). Approximately 20 horizontal head impulses were manually applied to each side with unpredictable timing and direction. Gain of HVOR was quantified at similar head acceleration in the both groups. The HVOR gain values were separated according to the direction (toward the right or left) of the head impulse. A significant difference between the two sides has been reported in healthy subjects (30). For the PI group, the mean peak head velocity was 195 ± 21°/s (mean peak head acceleration: 3957 ± 283°/s^2^) for impulses toward the left side and was 189 ± 29°/s (mean peak head acceleration: 3841 ± 943°/s^2^) for impulses toward the right side. For the control group, the mean peak head velocity was 189 ± 17°/s (mean peak head acceleration: 3709 ± 447°/s^2^) for impulses toward the left side and was 188 ± 19°/s (mean peak head acceleration: 3658 ± 518°/s^2^) for impulses toward the right side.

The VOR gain was calculated using two methods. First, the method described by MacDougall et al. (31): VOR gain was calculated as the area under the desaccaded eye velocity curve divided by the area under the head velocity curve. Second, a method developed in our laboratory using a linear regression (slope method). The linear regression was computed in MATLAB using linear polynomial curve fitting (polyfit) of the eye velocity from the start of the head movement to the peak of the head velocity. Noise was reduced by a rectangular low-pass filter using the discrete Fourier transform at 20 Hz for head velocity and at 38 Hz for eye velocity. Only data following almost perfectly a straight line were included in the analysis (linearity >98%). The difference between left and right sides was quantified as a gain asymmetry ratio: ratio = (L − R)/(L + R) × 100 where L and R are the mean gain values from the left and right head impulses, respectively.

### Cervical and Ocular VEMPs

Vestibular-evoked myogenic potentials were recorded with a Nicolet Viking 4 apparatus (Nicolet Biomedical Inc., Madison, WI, USA) with a four-channel averaging capacity, as previously described (32–34).

Cervical vestibular-evoked myogenic potentials assess predominantly the function of the sacculo-spinal pathways (35). They were recorded from surface electrodes above the tensed sterno-cleido-mastoideus (SCM) muscle ipsilateral to the stimulated ear in response to air-conducted (AC) short tone burst (STB) stimuli: 500 Hz, 102 dB nHL, 128 dB SPL, rise/fall time 2 ms, plateau time 2 ms, presented through calibrated TDH39 headphones. EMG activity of the SCM was monitored on a display to ensure that sufficient muscle activation was maintained (>150 μV).

Ocular vestibular-evoked myogenic potentials assess predominantly the function of the utriculo-ocular pathways (36). They were recorded from surface electrodes above the inferior oblique extraocular muscle contralateral to the stimulated ear in response to AC STB, and to bone-conducted vibration (BCV) at Fz, and to BCV stimulation at the mastoid. The AC STB (500 Hz, 110 dB nHL, 132 dB SPL, rise/fall time 2 ms, plateau time 2 ms) were presented through calibrated TDH39 headphones. BCV stimuli (500 Hz STB, 135 dB FL, rise/fall time = 2 ms and plateau time = 2 ms) were delivered by a hand-held Bruel and Kjaer (Naerum, Denmark) Mini-Shaker 4810.

Patients with no measurable response on either side were considered to be non-responders (NR).

### Equitest

Equilibrium was assessed by the Sensory Organization Test (SOT) on the EquiTest^®^ (37, 38). The SOT included six conditions. *Condition 1*: the subject was asked to stand upright while maintaining eyes open. *Condition 2*: the subject was asked to stand upright while maintaining eyes closed. *Condition 3*: the cabin moved adaptively following subject’s movements. In this condition, the vision was sway-referenced. *Condition 4*: the support base moved adaptively following subject’s movements while eyes were open: sway-referenced proprioception. *Condition 5*: same as condition 4, but with eyes closed. *Condition 6*: the support base and the cabin moved in a synchronized way: vision and proprioception are sway-referenced. According to the change of the body center of pressure for the six different conditions, a percentage somatosensory, visual, and vestibular score was calculated, a visual preference was estimated, and a composite score was obtained.

### Vibration-Induced Nystagmus and Head-Shaking Nystagmus Test

Spontaneous nystagmus was tested using an infrared camera with the subject in a sitting and a supine position. Vibration-induced nystagmus was tested with a vibratory stimulation of 100 Hz applied to the mastoid (39–41). Head-shaking stimulation consisted of turning the head of the patient in the horizontal plane to the left and the right at 2 Hz for 20 s (42–44). The presence of nystagmus during one or more of these tests indicated asymmetry of vestibular function between the ears in the horizontal plane.

### Audiometric Tests

Tympanometry and stapedial reflexes were carefully evaluated to exclude patients suffering from conductive hearing loss, which could lead to a misinterpretation of ACS VEMPs. The mean pure-tone threshold (PTA) for tones at 250 and 500 Hz and 1 and 2 kHz was used as indicator of hearing loss.

## Results

### Dizziness Handicap Inventory

The DHI score 39 ± 11% was for the PI group and 14 ± 19% for the control group. In the control group, 3 individuals out of 10 with high score (20, 22, and 30), the remaining individuals had DHI score inferior or equal to 10. These three patients did not complain for instability (and did not fail on the Equitest). These high score was linked to high scores at questions related to their difficulty at performing head movement (French DHI, questions 1, 8, 11, 12, 13, 25), a current syndrome without objective deficits in seniors over 70 years old. This difference was significant (non-parametric Mann–Whitney test, *p* = 0.002).

### Vestibular Horizontal Canal Receptor Function

Horizontal canal function was assessed using both video-nystagmoscopy and video-nystagmography. None of the study population (control or PI group) exhibited spontaneous nystagmus in darkness or ocular nystagmus induced by either head shaking or vibration.

#### Caloric Tests

No significant difference was detected between PI and control groups for the peak SPV of caloric ocular nystagmus induced either by warm or cold water irrigations (Table 1). No CP was detected in either group.

**Table 1:** **Mean slow-phase peak velocity (SPV) of the induced ocular nystagmus obtained for warm and cold irrigations in PI and control senior groups**.

SPV (°/s)	SPV left warm	SPV right warm	SPV left cold	SPV right cold
PI	25 ± 9.4 (15–40)	25 ± 5 (17–32)	13 ± 5 (7–19)	12 ± 4 (7–16)
Control	27 ± 8 (20–42)	24 ± 5 (15–33)	16 ± 5 (6–24)	15 ± 4 (11–23)

*There was no significant difference between the two groups*.

**Figure 2:**
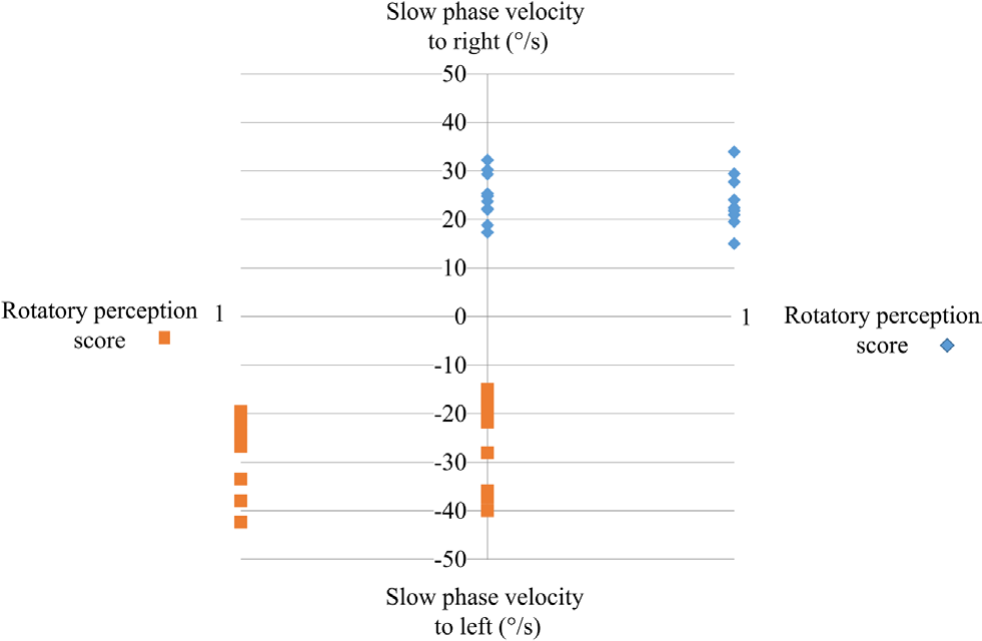
**The perception of rotation score during caloric testing as a function of the peak of the slow-phase velocity**. Ordinates: peak SPV induced by warm water irrigation of the right or the left ear; abscissa: perception rotation score during irrigation. Note that the seniors with postural instability did not perceive any rotation (score 0) irrespective of whether the left or right ear was irrigated; all controls perceived rotation.

All of controls perceived rotation (score 1) (Figure 2) when the maximal SPV during warm irrigation was ≥15°/s. The direction of the perceived rotation was in all cases toward the side of the fast phase of the induced ocular nystagmus. The perception of rotation increased progressively to a maximum at the peak of the SPV and then progressively decreased in good agreement with the eye velocity of the induced caloric nystagmus. The perception disappeared when eye velocity fell to or below 10°/s. In most cases, the patient’s head turned progressively in the direction of the slow component of caloric-induced eye nystagmus for both warm and cold irrigation.

None of the subjects in the PI group perceived rotation (score 0) (Figure 2) even while the peak SPV exceeded 15°/s. They all stated that did not feel anything: absolutely no sensation at all of head or body rotation.

#### Horizontal Video Head Impulse Test

**Figure 3:**
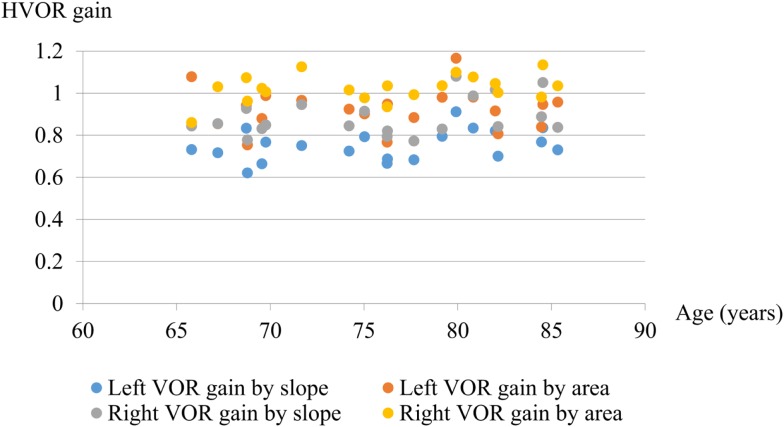
**Graph showing the gain of HVOR calculated by slope and area methods**. There was no significant difference in HVOR gain with age between 65 and 86 years even for high accelerations (mean 3700 ± 550°/s^2^).

There was no significant difference between the two groups in the mean HVOR gain calculated by either slope or area method (Table 2). The HVOR gain calculated using the slope method was similar to that using the area method, although the value obtained from the area was consistently greater that from the slope (Figure 3). The HVOR gain ratio for the PI group was: −8.3 ± 2.0 (min–max: −11.5 to −5.0), which was not significantly different from that for the control group: −8.9 ± 2.1 (min–max: −11.4 to −5.3).

**Table 2:** **Mean HVOR gain calculated with slope and area methods in PI and control seniors groups**.

hVOR gain	Left with slope	Left with area	Right with slope	Right with area
PI	0.76 ± 0.06	0.95 ± 0.11	0.90 ± 0.08	1.02 ± 0.07
Control	0.73 ± 0.08	0.90 ± 0.08	0.88 ± 0.09	1.02 ± 0.06

*There was no significant difference between the two groups*.

These investigations show for the first time that vestibular perception can be absent during a caloric test despite objective measures of horizontal canal functioning suggesting that it is normal. We went on to test whether vestibular perception can be associated with PI.

### Vestibular Otolithic (Utricular and Saccular) Receptor Function

None of our subjects exhibited conductive hearing loss. The mean PTA in the PI group was 30 ± 20 dB (min–max: 0–110 dB) and in the control group was 35 ± 25 dB (min–max: 5–110 dB).

#### Cervical VEMPs in Response to AC STB

The mean peak-to-peak (corrected and uncorrected) amplitudes of the early P13–N23 waves were not significantly different between the two groups (Table 3). The EMG activity of the SCM muscle was similar: 180 ± 42 for PI subjects versus 184 ± 70 for control subjects. No significant difference was found for the P13 and N23 latencies between the two groups. Forty percent of PI and 30% of the control group were NR.

**Table 3:** **Mean values of amplitude and latencies for cVEMPs and oVEMPs for PI senior and control senior groups in response to ACS and BCV at Fz and mastoid**.

		Amplitude (**μ**V)	P13/n1 latency (ms)	N23/p1 latency (ms)
ACS 102 dB cVEMPs	PI	Uncorrected: 79 ± 129	15.3 ± 0.9	22.3 ± 2.0
		Corrected: 0.41 ± 0.67		
	Control	Uncorrected: 85 ± 88	15.0 ± 1.1	21.4 ± 1.5
		Corrected: 0.56 ± 0.45		
ACS 110 dB oVEMPs	PI	2.1 ± 4.0*	11.4 ± 0.5	15.4 ± 1.0
	Control	5.9 ± 5.3	11.0 ± 0.4	14.6 ± 0.8
Fz BCV oVEMPs	PI	3.6 ± 5.0*	11.3 ± 0.8	15.6 ± 1.0
	Control	8.7 ± 7.7	11.0 ± 0.7	15.0 ± 0.8
Mastoid BCV oVEMPs	PI	7.7 ± 6.1	11.5 ± 1.0	15.7 ± 1.3
	Control	12.9 ± 9.8	10.8 ± 0.8	15.2 ± 0.8

*Non-parametric Mann–Whitney test, *p* = 0.001.

#### Ocular VEMPs in Response to AC STB

The mean peak-to-peak n1–p1 amplitude was significantly lower in the PI group than the control group (Table 3). There was no significant difference for the n1 and p1 latencies between the two groups. Sixty percent of PI subjects and 30% of the control group were NR.

#### Ocular VEMPs in Response to BCV at the Fz Location

The mean peak-to-peak amplitude was significantly smaller in the PI group than the control group (Table 3). There was no significant difference between the groups for the n1 and p1 latencies. Sixty percent of PI subjects and 20% of controls were NR.

#### Ocular VEMPs in Response to BCV at Mastoid Location

The mean peak-to-peak amplitude was not significantly different between the PI and control groups (Table 3). The n1 and p1 latencies did not differ significantly between the two groups. Twenty percent of PI subjects and 10% of controls were NR.

### Equitest

**Figure 4:**
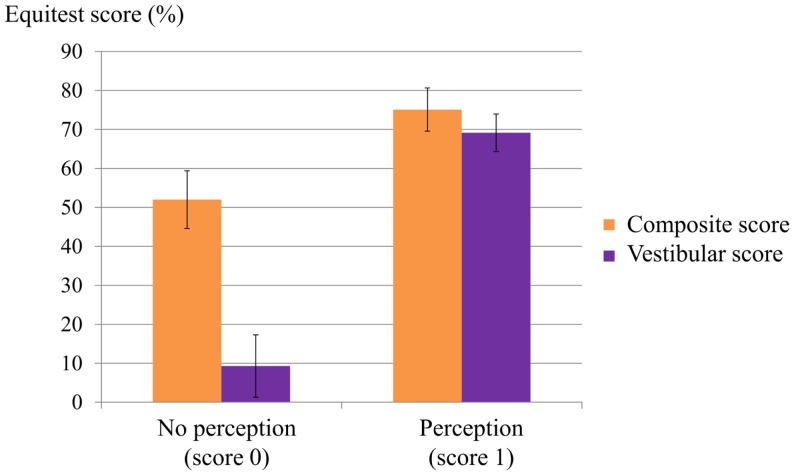
**Relation between composite and vestibular score on the Equitest and the perception score during caloric irrigation**. Most patients with a low perception score had a poor vestibular score on the Equitest and a low composite score.

All members of the control group had normal Equitest results in all six conditions. All the PI group had abnormal Equitest results: 80% fell in conditions 5 and 6; and 20% had a low score in condition 5 and fell in condition 6. Subjects with no perception of vertigo during the caloric test could not maintain balance in condition 5 (eyes closed, sway-referenced platform), a condition which tests the contribution of the vestibular inputs to balance (Figure 4).

## Discussion

In this work, we report that a subset of patient complaining of PI, when submitted to caloric irrigation, displayed a lack of perception of ego motion, contrasting with a normal ocular nystagmic response. Such vestibular neglect appearing quite uncommon, we assessed their full vestibular and postural performances. We found that poor vestibular perception in these 10 PI patients was associated with an inadequate postural strategy to maintain balance in conditions 5 (eyes closed + sway-referenced platform) and condition 6 (vision-referenced + sway-referenced platform) of the Equitest. We hypothesized that the absence of perception of movement during caloric test may be a marker of risk of fall which has not been considered before.

Our results are consistent with a study by Diard et al. (45). They reported that despite normal caloric tests, some seniors failed to maintain balance in the conditions 5 and 6 of the Equitest, a syndrome they called a vestibular omission. They suggested that this phenomenon was due to misapplication of normal peripheral vestibular information. However, they did not report whether patients perceived rotation during caloric stimulation.

Ours is the first report of a clear dissociation between horizontal canal function and perception of rotation during caloric stimulation in elderly subjects. We propose to call this dissociation “vestibular neglect.” Despite normal responses to warm caloric stimulation, the subjects have no perception of rotation and no perception of eye movements. The only similar report we are aware is by Takeda et al. (46), who describe a stroke patient with normal caloric responses and no perception of rotation during caloric stimulation. We found a relationship between vestibular neglect and PI. This result suggested a deficit of the central processing of vestibular information in patients exhibiting vestibular neglect. In summary, we suggest that a lack of egomotion perception during caloric test should draw the attention to PI and encourage measures to prevent falls. That said, it is clear that “vestibular neglect” may be one of the many causes of PI.

The effect on age on vestibular perception has been the subject of several studies. Roditi and Crane (17) used sinusoidal acceleration for surge (forward–backward), sway (left–right), heave (up–down), and yaw rotation. Only the thresholds for surge and sway for sinusoidal rotation at 0.5 and 1 Hz were found to be significantly higher in subjects >50 years old. Chang et al. (18) failed to detect any correlation between vestibular perception threshold, gain of VOR, and age using a rotational chair. A correlation has been found between horizontal perceptual threshold and oVEMP amplitude in the otolith system. In contrast, no significant association was detected for vertical perceptual thresholds and cVEMP amplitudes (10, 11). Therefore, vestibular perception is usually tested with more specific and quantified tests using rotatory chairs at various frequencies of head accelerations. In contrast, the caloric test imposes a large vestibular stimulation, activating all sensors at low frequencies. To our knowledge, such “brute force” was, rightly so, never employed to test vestibular perception. It may be useful to help to prevent fall but it cannot be considered as a bona fide test of vestibular perception.

The persistence of a normal HVOR in the PI group contrasted with the absence of perception of illusory movements during the caloric tests. Three non-mutually exclusive factors could be at play.

First, this dissociation can be explained by the differences between the vestibulo-ocular and the vestibulo-cortical and vestibulo-subcortical pathways. A trisynaptic pathway links the canal sensors to the extraocular motoneurons in 6 ms. In contrast, a polysynaptic, distributed network underlies self-motion perception: its first relay is in the vestibular nuclei, the second in the thalamus, and it includes several inter-connected cortical areas. These areas include the PIVC, temporal superior gyrus, inferior parietal lobe, and insula (47–49), where visual, vestibular, and proprioceptive inputs converge. A number of vestibular and cerebellum connections have been reported [for review see Ref. (50)] so a dysfunction of the cerebellum could be involved. Such complex circuitry may be more sensitive to the aging process that the three-neuronal arc of the VOR. Functional MRI during caloric stimulation may be informative and establish whether there is a link between the absence of perception of rotation and either a cognitive failure or abnormal activation of the areas devoted to integrate vestibular inputs at the subcortical level and/or the cortical network such as PIVC (48).

Second, the HVOR apparently does not decline with age as has been shown using calorics (8, 51) and vHIT: McGarvie et al. (9) failed to detect any significant decline of HVOR gain until age 90 years. Only a slight decrease of vertical VOR gain after age 80 years was found when head impulses were delivered in the plane of the posterior canal. These functional studies contrast with morphological data. In humans, the number and the density of hair cells decrease with age in the cristae vestibular ampulla between 60 and 90 years (52–54). Moreover, there are morphological changes to the cristae hair cell cilia, including a reduction of the numbers, disarrangements, and formation of giant cilia (55, 56). Clearly, the vestibulo-ocular network displays sufficient plasticity to cope with these cellular alterations.

Third, an inappropriate integration of otolith and canal signals and central reweighting of sensory inputs related to motion detection could participate to vestibular neglect. In that regard, the finding of Agrawal et al. (10, 11) that perceptual thresholds for linear motion increased in subjects with utricular dysfunction is relevant. The occurrence of utricular dysfunction augmenting with age, and it would lead to inappropriate integration of otolith and canal signals at the second order vestibular neurons level and consequently to misperception of the canal information induced by the caloric irrigation. We intend to test the hypothesis of an inappropriate integration of otolith and canal signals and central valuing by comparing the perceptual thresholds for linear motion of patients with and without vestibular neglect.

## Conclusion

We show that some seniors are unable to detect and report a subjective sensation of rotation during strong unilateral horizontal canal stimulation, despite objective evidence that these seniors have normal peripheral horizontal semicircular canal function. We suggest that this dissociation between perception and objective vestibular responses may be a determinant of PI, because it is these same seniors who demonstrate greater PI than age-matched controls. Therefore, failure to perceive rotation during caloric testing when the SPV is >15°/s should encourage the clinician to envisage preventive actions to avoid future falls such as rehabilitation. Further studies are needed to evaluate the proportion of PI seniors without vestibular perception, amongst a larger population of patients with complaints of PI.

## Author Contributions

CW and GL devised the protocol and wrote much of the paper; EC tested subjects, wrote much of the paper, and conducted the analyses; IC contributed to the redaction of the paper and consulted; CM made the Matlab program for vHIT data analysis; and P-PV reviewed the paper.

## Conflict of Interest Statement

Ian S. Curthoys is unpaid consultant to and has received funding for travel from GN Otometrics. The other co-authors declare that the research was conducted in the absence of any commercial or financial relationships that could be construed as a potential conflict of interest.

## Abbreviations

ACS, air-conducted sound; BCV, bone-conducted vibration; cVEMP, cervical vestibular-evoked myogenic potentials; DHI, Dizziness Handicap Inventory; H-VHIT, horizontal video head impulse test; HVOR gain, horizontal vestibulo-ocular reflex; NR, not responders to VEMPs; oVEMP, ocular vestibular-evoked myogenic potentials; PI, postural instability; PIVC, parieto-insular vestibular cortex; SCM, sterno-cleido-mastoideus; SOT, Sensory Organization Test; SPV, slow-phase velocity of the ocular nystagmus; STB, short tone burst; VEMPs, vestibular-evoked myogenic potentials; VHIT, video head impulse test; VOR, vestibulo-ocular reflex.
